# Menin-mediated regulation of miRNA biogenesis uncovers the IRS2 pathway as a target for regulating pancreatic beta cells

**DOI:** 10.18632/oncoscience.79

**Published:** 2014-09-15

**Authors:** Buddha Gurung, Bryson W. Katona, Xianxin Hua

**Affiliations:** ^1^ Abramson Family Cancer Research Institute, Department of Cancer Biology, University of Pennsylvania Perelman School of Medicine, 421 Curie Blvd., BRB II/III, Philadelphia, PA-19104, USA; ^2^ Division of Gastroenterology, University of Pennsylvania Perelman School of Medicine, 421 Curie Blvd., BRB II/III, Philadelphia, PA-19104, USA

**Keywords:** Menin, ARS2, miRNA processing, IRS1, Let7a, beta cell

## Abstract

Menin, a protein encoded by the *MEN1* gene, is mutated in patients with multiple endocrine neoplasia type 1 (MEN1). Menin acts as a tumor suppressor in endocrine organs while it is also required for transformation of a subgroup of leukemia. The recently solved crystal structure of menin with different binding partners reveals that menin is a key scaffold protein that cross-talks with various partners, including transcription factors, to regulate gene transcription. Our recent findings unravel a previously undiscovered mechanism for menin-mediated control of gene expression via processing of certain microRNA’s, thus adding to the plethora of ways in which menin regulates gene expression. By interacting with ARS2, an RNA binding protein, menin facilitates the processing of *pri-let 7a* and *pri-miR155* to *pre-let 7a* and *pre-miR155* respectively. Consistently, excision of the *Men1* gene results in upregulation of IRS2, a *let-7a* target. As IRS2 is known to mediate both insulin signaling and insulin-induced cell proliferation, and *let-7a* targets include oncogenes like RAS and HMGA2, a deeper understanding of the menin-ARS2 complex in regulating miRNA biogenesis will yield further insights into the pathogenesis of the MEN1 syndrome and other menin-associated malignancies.

Multiple endocrine neoplasia type 1 (MEN1) is an inherited syndrome that is characterized by the development of neuroendocrine tumors in the parathyroid glands, pancreatic islet cells, anterior pituitary gland, and other sites such as the duodenum [1]. The gene mutated in this syndrome, *MEN1*, is located on chromosome 11q13 and encodes the primarily nuclear protein, menin [2]. A germline *MEN1* mutation, which is also often detected in sporadic pancreatic neuroendocrine tumors [3], can lead to the development of familial MEN1 in an autosomal dominant fashion. While biochemical tests have suggested a prevalence of the MEN1 syndrome at 0.01–0.175 per thousand [4, 5], the incidence of the syndrome based on genetic testing of the *MEN1* gene in a large population is not yet clear. Since the original discovery of the *MEN1* gene in 1997 [2], extensive work from multiple groups has been devoted toward understanding the biochemical functions of the *MEN1* encoded protein, menin, as well as its underlying mechanisms of action. The recently solved crystal structure of menin demonstrates that it acts as a scaffold protein in regulating gene transcription, cell proliferation, apoptosis and genome stability vis-à-vis its interaction with various protein partners [6-10]. However, the precise mechanism by which menin mediates these functions remains to be further explored. Excellent reviews on the molecular mechanisms whereby menin controls gene transcription, proliferation, and apoptosis have previously been published [11-16].

## Defining a novel role for menin as a posttranscriptional regulator of miRNA

Recently, we identified ARS2 (arsenite-resistance protein-2) as a new menin-binding partner [17]. ARS2 is a component of the nuclear RNA cap-binding complex that stabilizes certain primary microRNA (*pri-miRNA*) transcripts for processing by the Microprocessor complex consisting of Drosha and DGCR8, thereby playing an important role in miRNA-mediated gene silencing [18, 19]. We further demonstrated that the menin-ARS2 complex functionally controls the processing of *prilet 7a* [17]. This is of importance since the *let-7* family of microRNAs, which was first discovered in *Caenorhabditis elegans* as a key developmental regulator, has significantly decreased expression in human cancers and cancer stem cells with elevated levels of oncogenes including RAS and HMGA2 [20-23]. Furthermore, the *lin28/let 7* pathway acts as a central regulator of mammalian glucose metabolism and insulin signaling therefore implicating this pathway in diabetes mellitus [24, 25].

To specifically examine processing of *pri-let 7a*, we demonstrated that acute excision of the *Men1* gene results in reduced levels of certain miRNAs including *let- 7a* and *miR-155*, and this reduction was due to impaired processing of *pri-let 7a* to *pre-let-7a* [17]. However, levels of the pri-miRNA transcripts, including *pri-let7a* and *pri-miR155*, are not affected by menin, indicating that menin-mediated regulation does not occur at the level of transcription. Unlike menin’s previously described role in regulating gene expression at the level of gene transcription via epigenetic histone modifications or direct transcriptional activation [26], this is the first report demonstrating menin’s role in regulating target gene expression at the post-transcriptional level. Through menin’s binding to ARS2, this data also highlights menin’s role as a scaffold protein and its versatility in regulating gene expression via association with its multiple binding partners.

We subsequently explored the mechanism whereby menin post-transcriptionally regulates the levels of these specific miRNAs. In assays using **^32^**P-radiolabeled *prilet7a* as the substrate, we found that menin is required for processing of *pri-let7a* to *pre-let7a*. However, menin does not affect the levels of Drosha, an RNase III enzyme that executes the initial step of the miRNA processing in the nucleus [27]. This finding indicates that menin likely enhances the stability of the *pri-let7a* transcript and subsequent delivery of the *pri-let7a* transcript to the catalytic Microprocessor complex. Through targeted gene knockdown analysis, the interaction between menin and ARS2 was found essential for processing of *pri-let7a*, as *Ars2* knockdown reduced mature *let-7a* levels in meninexpressing cells, but not in menin-null cells. It was recently reported that in addition to its involvement in the biogenesis of miRNAs, ARS2 also regulates a number of mRNAs, in particular histone-encoding mRNAs by contributing to histone mRNA 3′ end formation and facilitating its cleavage [28]. Given the role of menin in epigenetic regulation of target gene expression is well characterized [7, 9, 29], it would be of interest to evaluate whether menin regulates certain genes by facilitating both modification of histone tails as well as histone expression in conjunction with ARS2.

## Elucidating the role of menin-mediated let-7a processing in regulating beta cell proliferation and insulin signalling

**Figure 1:**
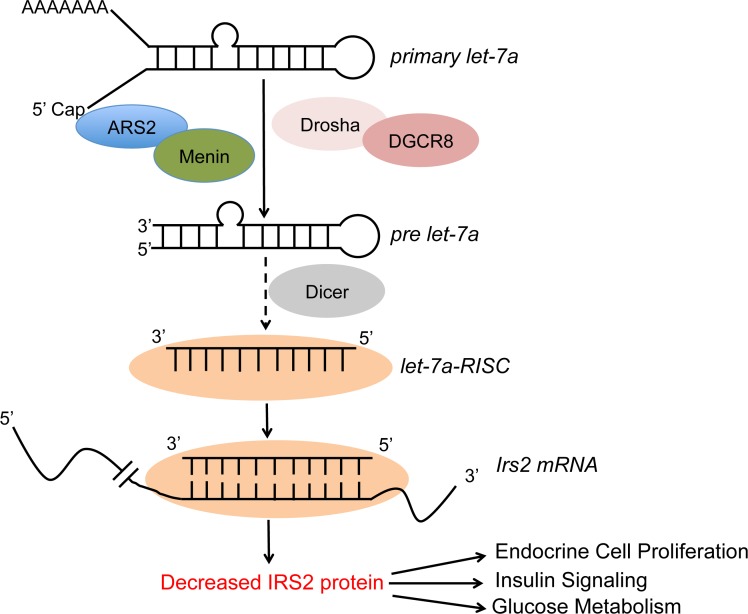
A model for menin/ARS2-mediated processing of Let-7a in regulating endocrine cell proliferation, insulin signaling, and glucose metabolism

Using prediction algorithms, IRS2, a protein involved in the insulin-signaling pathway, was previously reported as a *let-7a* target based on sequence complementarity analysis [25]. Furthermore, inhibition of let*-7a* prevents the down-regulation of IRS2 in the liver of mice on a high-fat diet, highlighting *Irs2* as a *bona fide* target of *let-7a* [25]. Physiologically, IRS2 has been shown to play a central role in peripheral insulin signaling and pancreatic beta cell proliferation, as knockout of *Irs2* in mice results in resistance to insulin and the development of type 2 diabetes [30]. We have previously shown that excision of the *Men1* gene ameliorated pre-existing hyperglycemia and increased both glucose-stimulated insulin release and circulating insulin levels in mouse diabetes models [31]. As such, we set to explore a possible link between menin’s role in *let-7a* processing and its overall role in controlling pancreatic beta cell proliferation and function. These studies demonstrate that meninexcision led to increased IRS2 expression. Furthermore, ectopic expression of menin in insulin-producing βHC9 cells, which are derived from pancreatic islets with beta cell hyperplasia, and BON cells, a carcinoid cell line, results in decreased levels of IRS2. Our findings thus suggest that the elevated levels of *let-7a* in meninexpressing cells targets the *Irs2* mRNA for degradation, and the excision of the *Men1* gene subsequently relieves this targeted mRNA degradation, resulting in enhanced IRS2 expression. Additionally, we showed that anti-miRmediated knockdown of *let-7a* results in a significant increase of IRS2 in menin-expressing cells but not in menin-null cells. These findings clearly indicate that the decreased levels of IRS2 in menin-expressing cells, at least partly, result from the elevated levels of *let-7a* (Figure 1).

In line with decreased levels of *let-7* in various tumors, and oncogenes (including RAS) amongst *let- 7* target genes, it is plausible that menin’s role in tumor suppression can be attributed, in part, to enhanced levels of *let-7a* biogenesis via its interaction with ARS2 [21, 22]. Accordingly, it has been shown that reduced menin expression is associated with enhanced expression of Ras in human lung adenocarcinoma samples [32]. Examining whether increased Ras expression in lung adenocarcinoma harboring reduced menin levels correlates with diminished *let-7a* biogenesis might yield some new insight into the pathogenesis of the disease. Furthermore, *miR-155*, whose biogenesis is also regulated by menin [17], was among the top down-regulated miRNA’s in pancreatic neuroendocrine tumors, further implicating the role of miRNA’s in menindependent repression of cell proliferation [33]. Similarly, the biogenesis of several other microRNAs, in addition to *let-7a* and *miR-155*, in the presence of menin might collectively contribute to its role in tumor suppression. In this regard, a detailed comparison analysis of differential miRNA expression in menin-null cells compared to menin wild-type cells will shed light on the identities of miRNAs whose biogenesis is possibly regulated by the menin-ARS2 pathway thereby providing deeper insight into menin-mediated repression of cell proliferation.

## Exploiting the menin-ARS2 axis to understand the mechanism of MEN1 tumorigenesis and beta cell regeneration

A detailed understanding of the menin-ARS2 interaction and its effects on beta cell proliferation via miRNA biogenesis might yield valuable insight into the mechanisms by which loss of menin function results in MEN1 tumor syndrome. It has previously been shown that *let-7* expression is reduced in a population of human lung cancers, and furthermore its decreased expression is associated with shortened post-operative survival [34]. Similarly, decreased levels of *let-7a*, in part, could lead to MEN1 tumorigenesis as a result of elevated levels of target oncogenes including RAS. Examining the global levels of *let-7a* in MEN1 patients would provide important clues as to whether menin-associated loss of *let-7a* processing plays a role in the pathogenesis of the disease. Importantly, microarray analysis of the expression profile of miRNAs in menin-containing and menin-null cells would uncover other miRNAs in addition to *let-7a* and *miR-155* whose processing is regulated by the menin-ARS2 complex, possibly implicating a larger role for menin-mediated miRNA processing in tumorigenesis.

It has previously been shown that the global knockdown of *let-7* was sufficient to prevent as well as ameliorate impaired glucose tolerance in mice on a high fat diet [25]. As a corollary to menin-ARS2 complex repressing *Irs2*, a key player in regulating beta cell mass and function [35], the discovery of a small molecule that interferes with this binding may hold considerable promise for use in the treatment of type 2 diabetes.
